# A Wearable Artificial Intelligence Feedback Tool (Wrist Angel) for Treatment and Research of Obsessive Compulsive Disorder: Protocol for a Nonrandomized Pilot Study

**DOI:** 10.2196/45123

**Published:** 2023-07-24

**Authors:** Nicole Nadine Lønfeldt, Line Katrine Harder Clemmensen, Anne Katrine Pagsberg

**Affiliations:** 1 Child and Adolescent Mental Health Center Mental Health Services CPH Copenhagen University Hospital Hellerup Denmark; 2 Department of Applied Mathematics and Computer Science Technical University of Denmark Lyngby Denmark; 3 Department of Clinical Medicine Faculty of Health and Medical Sciences University of Copenhagen Copenhagen Denmark

**Keywords:** machine learning, signal processing, wearable biosensor, obsessive compulsive disorder, oxytocin, children, adolescents, family accommodation, psychiatric symptoms, clinical practice, automatic assessment tool, psychotherapy, mental health

## Abstract

**Background:**

Obsessive compulsive disorder (OCD) in youth is characterized by behaviors, emotions, physiological reactions, and family interaction patterns. An essential component of therapy involves increasing awareness of the links among thoughts, emotions, behaviors, bodily sensations, and family interactions. An automatic assessment tool using physiological signals from a wearable biosensor may enable continuous symptom monitoring inside and outside of the clinic and support cognitive behavioral therapy for OCD.

**Objective:**

The primary aim of this study is to evaluate the feasibility and acceptability of using a wearable biosensor to monitor OCD symptoms. The secondary aim is to explore the feasibility of developing clinical and research tools that can detect and predict OCD-relevant internal states and interpersonal processes with the use of speech and behavioral signals.

**Methods:**

Eligibility criteria for the study include children and adolescents between 8 and 17 years of age diagnosed with OCD, controls with no psychiatric diagnoses, and one parent of the participating youths. Youths and parents wear biosensors on their wrists that measure pulse, electrodermal activity, skin temperature, and acceleration. Patients and their parents mark OCD episodes, while control youths and their parents mark youth fear episodes. Continuous, in-the-wild data collection will last for 8 weeks. Controlled experiments designed to link physiological, speech, behavioral, and biochemical signals to mental states are performed at baseline and after 8 weeks. Interpersonal interactions in the experiments are filmed and coded for behavior. The films are also processed with computer vision and for speech signals. Participants complete clinical interviews and questionnaires at baseline, and at weeks 4, 7, and 8. Feasibility criteria were set for recruitment, retention, biosensor functionality and acceptability, adherence to wearing the biosensor, and safety related to the biosensor. As a first step in learning the associations between signals and OCD-related parameters, we will use paired *t* tests and mixed effects models with repeated measures to assess associations between oxytocin, individual biosignal features, and outcomes such as stress-rest and case-control comparisons.

**Results:**

The first participant was enrolled on December 3, 2021, and recruitment closed on December 31, 2022. Nine patient dyads and nine control dyads were recruited. Sixteen participating dyads completed follow-up assessments.

**Conclusions:**

The results of this study will provide preliminary evidence for the extent to which a wearable biosensor that collects physiological signals can be used to monitor OCD severity and events in youths. If we find the study to be feasible, further studies will be conducted to integrate biosensor signals output into machine learning algorithms that can provide patients, parents, and therapists with actionable insights into OCD symptoms and treatment progress. Future definitive studies will be tasked with testing the accuracy of machine learning models to detect and predict OCD episodes and classify clinical severity.

**Trial Registration:**

ClinicalTrials.gov NCT05064527; https://clinicaltrials.gov/ct2/show/NCT05064527

**International Registered Report Identifier (IRRID):**

DERR1-10.2196/45123

## Introduction

### Background

Up to 3% of children and adolescents suffer from obsessive compulsive disorder (OCD) [1], which is characterized by repetitive, intrusive thoughts about threats of contamination, harm to self or others, loss of esteem, and committing moral transgressions, accompanied by repetitive or ritualized behaviors that can be time consuming, such as cleaning, checking, and ordering [2]. These obsessive thoughts and compulsive behaviors lead to marked distress, avoidance, and negative emotion (eg, anxiety and disgust) [2]. To regulate OCD-related distress, youths (individuals below the age of 18 years) may avoid situations that trigger obsessions and compulsions, perform rituals to neutralize feelings of incompleteness or magically remove the threat, or seek reassurance and help from others such as parents [3]. Parents are biologically primed to be sensitive to distress signals from their children [4,5]. Thus, parents may become distressed when their children are distressed. Interactions between parents and youths with OCD can be conflictual when parents refuse to accommodate their children’s symptoms [6]. Youths may coerce or use verbal and physical aggression to elicit accommodation or parents may voluntarily provide family accommodation to regulate their own or their children’s distress [7]. Family accommodation refers to ways in which parents help their children with OCD perform rituals, avoid stimuli that trigger symptoms, and change their own routines because of their children’s OCD symptoms [8]. This reciprocal coordination of parent-child behavior is reflected in the parent’s and child’s physiology and may in part be facilitated by the oxytocin system [9]. In short, OCD is characterized by distress, negative emotions, and behavioral repertoires in individual youths and their parents that can be directly observed or indirectly detected in behavior, facial expressions, speech patterns, and physiological signals.

Automatic, continuous detection of these OCD signals would facilitate close monitoring of patient treatment progress and allow for more targeted interventions. The first-line treatment for OCD is cognitive behavioral therapy with exposure and response prevention (ERP) [10,11]. Continuous monitoring of distress levels and the function of distress during the treatment is essential for optimizing ERP [12]. Currently, patients are asked to rate their level of distress throughout an ERP task. Machine learning (ML) models that detect distress levels would automate distress monitoring and enable passive collection of objective measures allowing therapists to adjust exposures accordingly. Automatic, continuous distress detection may also facilitate the monitoring of treatment response across therapy sessions as well as monitoring disorder progression in individuals with subclinical symptoms. Finally, an important part of therapy is increasing awareness of the connections between thoughts, emotions, behaviors, and bodily sensations [13]. ML models that detect changes in mental states would facilitate increasing awareness of these connections.

Previous studies have demonstrated that ML models can detect physiological, behavioral, and speech signals that may be relevant to youth with OCD and thus aid in developing objective, intelligent monitoring tools that will enable timely actionable insights and interventions supporting psychotherapy in and outside of the clinic. A previous study demonstrated that ML models using 3 minutes of physiological data from a wearable biosensor worn by 20 youths could predict aggressive behaviors in youths with autism spectrum disorders 1 minute prior to their occurrence with relatively high accuracy [14]. Another study linked behavioral synchrony between 15 fathers and their 2-5–year-old children with Down syndrome to synchrony in the dyads’ electrodermal activity (EDA) measured with wearable biosensors during a play session [15]. Using speech features as input, ML models have classified the severity of depression [16] and differentiated between children with and without internalizing disorders [17]. ML models that use speech features as input have also been utilized to gauge the quality of interpersonal interactions in a psychotherapy setting [18]. Finally, ML models (ie, computer vision models) are also capable of analyzing data from videos to track movement; infer emotion from facial expressions; and estimate visual attention, engagement, and interpersonal interaction from gaze-tracking information [19,20]. A systematic review showed that computer vision studies have sample sizes as low as 2 participants, often in the range of 10-20 and with more than 50 participants in rare cases, to demonstrate the capabilities of computer vision as a clinical tool [19]. To date, no artificial intelligence (AI) tools for monitoring clinically relevant parameters of OCD in youth are available and validated. Thus, Wrist Angel aims to develop AI tools capable of predicting OCD episodes, related (mal)adaptive parent behavior, and continuously monitoring OCD-related distress in youths and parents.

### Goal of This Study

The purpose of this protocol is to provide a detailed account of our study design and methods for data collection. The primary aim of the Wrist Angel pilot study is to test the feasibility of using a wearable biosensor to detect OCD-related events and monitor clinical severity in children and adolescents. Ultimately, we aim to develop algorithms for detecting OCD-relevant parameters using physiological, audio, and visual signals as input and assess their accuracy. Thus, important information from the pilot study will be whether it is feasible to collect enough data to develop such algorithms. Specifically, we will test the feasibility of recruiting and retaining participants for brief experiments in the clinic and an 8-week observational study during which youths and parents wear a biosensor in their everyday lives. Feasibility will also be assessed in terms of biosensor safety and functionality, participant acceptance of the biosensor, and participant adherence to wearing the biosensor. We also explore methods for collecting ecological momentary assessments (EMAs) for labeling data collected in the 8-week observational study, including a mobile app for data collection. Our primary hypothesis is that the study protocol will demonstrate sufficient feasibility to support subsequent larger studies.

Biosensors are worn by youths and one of their parents, as OCD causes distress in youths with the diagnosis as well as their parents. Furthermore, efficacious psychotherapy approaches for OCD in youth intervene by working either exclusively with the child [13] or the parent [21]. We also include measurements of oxytocin levels and response in youths and parents, as oxytocin may hold important information about how family dynamics interact with OCD symptoms and the optimal type of psychotherapy to address these interactions [22].

## Methods

### Design

The reporting of this protocol follows the recommendations for reporting protocols of pilot and feasibility trials [23]. We used the SPIRIT (Standard Protocol Items: Recommendations for Interventional Trials) 2013 checklist for interventional trials [24] ignoring nonapplicable items, supplementing with relevant items from the CONSORT (Consolidated Standards of Reporting Trials) extension to pilot trials [25] and making relevant modifications according to the guidelines for reporting nonrandomized pilot and feasibility studies [26] (see Multimedia Appendix 1).

**Figure 1:**
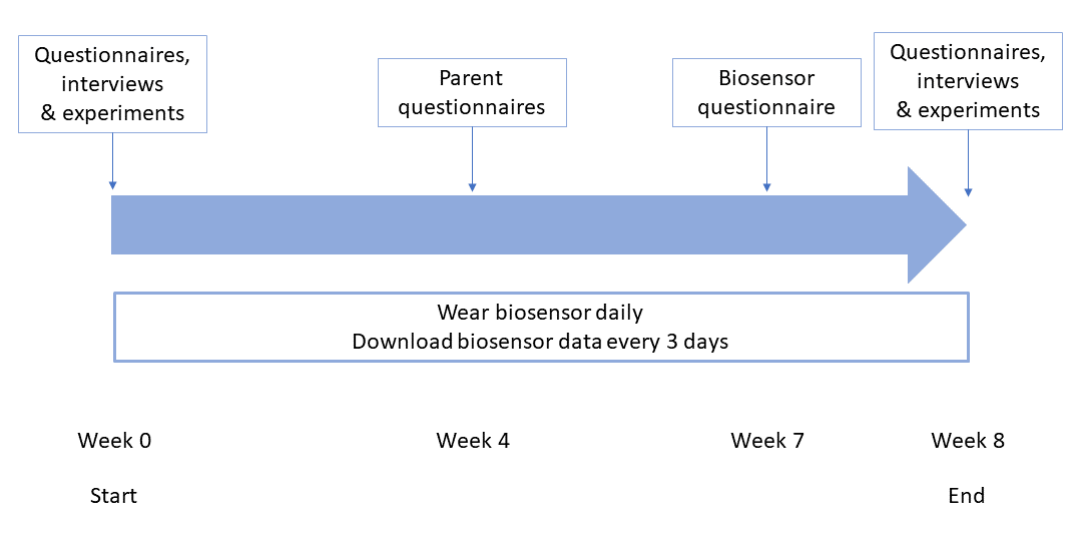
Design of the Wrist Angel pilot study. Lab visits were performed at week 0, or the start of the study (baseline), and at week 8. Participants completed self-rated questionnaires at weeks 0, 4, 7, and 8. Participants wore the biosensor for up to 8 weeks.

This is a nonrandomized, longitudinal pilot study, which includes a case-control comparison and repeated experiments. Youths and their participating parent are asked to wear a biosensor daily during waking hours for 8 weeks. In comparison, previous studies have collected data from 7 hours to 10 days [14]. To better understand these *in-the-wild* signals, we collect physiological signals and additional audio and visual signals under controlled conditions in the clinic. Figure 1 depicts the study design.

### Setting

This pilot study is being conducted at a public child and adolescent mental health center within a psychiatry department of a university hospital in Denmark, covering the geographical area of the Capital Region.

### Participants

We aim to enroll 10 patient-parent dyads and 10 healthy control-parent dyads. A small sample size was chosen for practical and economic reasons to establish proof of concept. We aim to collect a larger number of observations for each individual (8 weeks) compared to previous studies (less than 2 weeks) to assess feasibility within subjects in contrast to across subjects. The inclusion and exclusion criteria are summarized in Textbox 1.

**Participant inclusion and exclusion criteria.**

Patient inclusion criteria

- 8 to 17 years old (both inclusive)
- Primary or secondary diagnosis of obsessive compulsive disorder (OCD; International Classification of Diseases [ICD]-10 F42) [27] as set by a child and adolescent psychiatric team
- Primary psychiatric diagnosis that a psychiatrist determined is eligible for care within psychiatry
- Children’s Yale-Brown Obsessive-Compulsive Scale (CY-BOCS) [28] entry total score >7 points
- Normal intellectual functioning as indicated by parent or youth report, clinical observation, and/or the appropriate full-scale Wechsler Intelligence Scale (for Children-Fifth Edition or Wechsler Adult Intelligence Scale-Fourth Edition) [29,30] (Intelligence Quotient>70)
- Signed informed consent from legal guardian(s)

Patient exclusion criteria

- Disorders that contraindicate study participation, including substance dependence syndrome (ICD-10 F1x.2), schizophrenia/paranoid psychosis (ICD-10 F20-25 and F28-29), mania or bipolar disorder (ICD-10 F30 and F31), depressive psychotic disorders (ICD-10 F32.3 and F33.3), intellectual disorder (ICD-10 F70-79), pervasive developmental disorder (ICD-10 F84.0-84.4 and F84.8-84.9), not including Asperger syndrome [27]
- Any condition (eg, allergies, eczema, hypersensitivity associated with Asperger syndrome) that would prevent the child or parent from wearing a wristband biosensor
- Current participation in other OCD trials

Control participant inclusion criteria

- 8 to 17 years old (both inclusive)
- Lives in the catchment area of the study site during the study
- Normal intellectual functioning as indicated by parent or youth report, clinical observation, and/or the appropriate full-scale Wechsler Intelligence Scale (for Children-Fifth Edition or Wechsler Adult Intelligence Scale-Fourth Edition) [29,30] (Intelligence Quotient>70)
- Signed informed consent from legal guardian(s)

Control exclusion criteria

- Any current or previous psychiatric or intellectual disorders according to ICD-10 criteria, as assessed by Schedule for Affective Disorders and Schizophrenia for School-Age Children-Present and Lifetime Version (K-SADS-PL) [31] at the time of screening
- Current participation in other OCD trials
- Any condition (eg, allergies, eczema) that would prevent the youth from wearing a biosensor on the wrist

Parent inclusion criteria

- Has a child participating in the current study
- Signed informed consent

Parent exclusion criteria

- Any condition (eg, allergies, eczema) that would prevent them from wearing a biosensor on the wrist

### Concomitant Care

There are no prohibited forms of care or interventions. Patients could be on wait lists, in any form of psychotherapy, or taking any form of pharmacological treatment.

### Recruitment

Patient participants are recruited from patients referred to the child and adolescent mental health center with probable OCD symptoms or a set OCD diagnosis. Potential patient participants’ journals are first screened for eligibility. Case workers then ask patient families if our research group may contact them about our research project. The control group is recruited from the control group of the Treatment Effects of family based Cognitive Therapy in children and adolescents with Obsessive compulsive disorder (TECTO) trial [32] and schools in the catchment area. After obtaining permission from the school administration, flyers are posted in the school or the school intranet.

Psychologists, medical doctors, and masters-level medical and psychology students screen and assess potential participants. For previous TECTO participants, we use their clinical information from the TECTO trial to determine eligibility. Potential control participants who contact us are screened for intellectual disability and psychiatric diagnoses using the Schedule for Affective Disorders and Schizophrenia for School-Age Children-Present and Lifetime Version (K-SADS-PL) criteria [31].

The recruitment period ranged from September 15, 2021, until December 31, 2022.

### Ethical Considerations

#### Approval and Consent

The Ethics Committee of the Capital Region of Denmark approved this study on June 17, 2021 (H-18010607-79689). This study is registered at ClinicalTrials.gov (NCT05064527; October 1, 2021). The interdisciplinary team of principal investigators (the authors) are responsible for the design and conduct of the study and preparation of the protocol and revisions. The principal investigators at the hospital are responsible for recruitment and monitoring participants. Any major modifications or protocol deviations are discussed with the other principal investigators during weekly meetings. Any major protocol modifications are reported to the Ethics Committee and amended on ClinicalTrials.gov.

After families assent, we inform the families about the project verbally, in person or over the telephone, and send written participant information via secure email. After receiving verbal and written information about the study, youth and parents are given 24 hours to deliberate. A psychologist or medical doctor obtains signed consent forms from legal guardians of youth and their participating parent along with assent from participants before participating in the study. A model consent form is available in Multimedia Appendix 2.

#### Safety

We are not aware of any major risks or safety issues associated with participation. Adverse reactions to the biosensor are monitored throughout the study (see Multimedia Appendix 1). Any serious adverse events will be reported to the sponsor and The Ethics Committee of the Capital Region of Denmark. Participants have the right to submit complaints and seek compensation for injuries or unsatisfactory treatment related to the study.

#### Compensation

Child and parent participants receive a DKK 600 (~US $90) gift card upon completion of their participation and return of the biosensor.

#### Withdrawal/Discontinuation From the Study

A participant who no longer wishes to participate in the study can withdraw their informed consent at any time without explanation nor consequences for their treatment. Consent can be withdrawn from parts of the study. For example, participants can consent to biosensor data collection and withdraw their consent for video data. Participants are also allowed to withdraw from parts of the pilot study such as the experiments, the 8-week observation period, and completion of measurements. We withdraw participants if they experience an untenable adverse reaction related to the biosensor or data collection procedures, they consistently do not attend data download meetings, the child participant receives a diagnosis included in our exclusion criteria, or we otherwise deem continued participation to be unsafe. We modify measurement requirements in the case of nonserious adverse events (eg, skin irritation at the site of the biosensor) and reduce burden of measurements if they are too frequent for the participant or found to be incomprehensible to the participant.

#### Data Management

Data collection, storage, and security adhere to the regulations set by The Knowledge Centre on Data Protection Compliance in The Capital Region of Denmark and follow the General Data Protection Regulation of the European Union. Signed data processing agreements between The Capital Region of Denmark and the Technical University of Denmark allow our interinstitutional research team to share data for analysis. Participating child-parent dyads are identified using a unique identification number with each dyad’s data linked to the unique identifier.

Original electronic, paper, and relevant medical records are source documents. The research team will ensure that all data entered in the central database represent a true record of events. Study data are collected and managed using Research Electronic Data Capture (REDCap) electronic data capture tools hosted at The Capital Region of Denmark [33,34]. REDCap is a secure, web-based software platform designed to support data capture for research studies, providing (1) an intuitive interface for validated data capture, (2) audit trails for tracking data manipulation and export procedures, (3) automated export procedures for seamless data downloads to common statistical packages, and (4) procedures for data integration and interoperability with external sources.

Empatica, the manufacturer of the E4 biosensor, is also a data processor. Physiological signals recorded by the E4 biosensor are stored on a central database managed by Empatica. Anonymized biosensor data, which can only be traced to our research group, are transferred to Empatica’s E4 Connect via a secure Amazon Web Service server. E4 Connect stores physiological signals under unique device identifiers. Our research group then downloads and stores physiological signal data on a logged drive of The Capital Region of Denmark where the data are connected to the unique participant identification number.

### Wearable Biosensor

Prior to the experiments at baseline, we instruct participants on how to use and care for the biosensor, which the youth and parent then place on their nondominant hand. Following the manufacturer’s manual, we inform the participants that they should not look directly at the light-emitting diode lights, that the biosensor tolerates splashes of water during handwashing and rain but not submersion, and that the biosensor should be stored at room temperature and out of direct sunlight. Participants are asked to charge the biosensor at night. We demonstrate how to put on the wristband, how to turn it off and on, how to tag events, and how to click the charging cradle on and off the wristband. All participants received an abridged user manual that we translated into Danish.

The E4 (Empatica, Milan, Italy) wristband is a US Food and Drug Administration (FDA)-approved Class II device for adults and children (FDA 2018, 2019) and a European Union Class IIa device denoting that it is a noninvasive monitoring device [35]. The E4 battery can last over 48 hours and can store up to 60 hours of data. The E4 wristband contains a photoplethysmographic (PPG) sensor, an EDA sensor, a 3-axis accelerometer, and an optical thermometer. PPG uses infrared light to measure changes in blood volume (ie, blood volume pulse) calculated by an algorithm that uses light signals during red and green exposure, sampled 64 times per second, as input [36]. The interbeat interval timings and heart rate are computed using the blood volume pulse signal as input to algorithms [36]. Under conditions of slight movement (<30% of the time), it is possible to use the interbeat interval to compute heart rate variability. The EDA sensor measures skin conductance sampled 4 times per second [37]. The accelerometer measures movement on three axes at a sampling rate of 32 times per second. The optical thermometer measures skin temperature at a sampling rate of 4 times per second [37].

### Data Collection

#### Assessments

The assessment time of each measure is outlined in Multimedia Appendix 3.

After recruitment (baseline assessment), we obtain information about mental health treatment history for the patients by asking the youths and parents and checking the youths’ medical journals. Medical journals are also used to obtain patient multiaxial diagnoses. During baseline testing, we ask participating parents to report probable or confirmed psychiatric diagnoses in the youths’ first-degree relatives, and we ask youths about their current stage of puberty using the Tanner stages [38]. After baseline testing, participating families are asked to complete a set of questionnaires online to provide demographic information and baseline measures of youth quality of life, family dynamics, and parent symptoms of affective disorders. The demographic questionnaire includes questions about age, sex, and country of origin for the youths and parents as well as parents’ highest attained level of education. Youths and parents also complete a questionnaire that includes questions about menstruation, hormone medication use, pregnancy, and breastfeeding. Information about hormones and puberty are collected as these are factors that may influence salivary oxytocin levels. Parents are also asked to complete the KIDSCREEN-52 [39], Parental Stress Scale (PSS) [40], Depression, Anxiety and Stress Scale (DASS) [41], and the Family Environment Scale [42]. Youths receive the KIDSCREEN-52 [39]. Parents of patients are also asked to complete a modified version of the Family Accommodation Scale–Parent Rated (FAS-PR) [43,44].

At the 8-week follow-up, patients’ OCD severity is assessed with the Children’s Yale-Brown Obsessive Compulsive Scale (CY-BOCS) before the experiments. After the experiments, all youth-parent dyads are interviewed for a maximum of 30 minutes about their experiences with the biosensor and their opinions about potentially using an app for data collection. Patient interviews are video- and audio-recorded. All participant answers are paraphrased and recorded in the REDCap database during the interview.

#### Experiments

**Figure 2:**
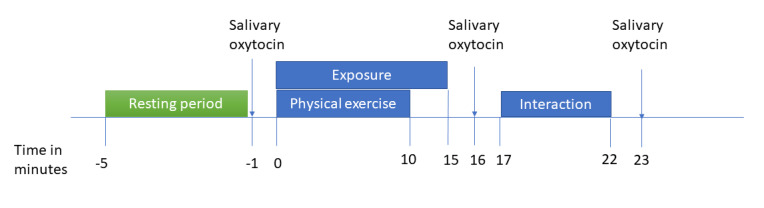
Experiment design. Participants complete experiments during lab visits at week 0 and 8. Before providing the first saliva sample, participants rest for 5 minutes. Afterward, patients complete an exposure task for up to 15 minutes and controls are physically active for about 10 minutes. All participants complete a parent-child interaction task that lasts for 5 minutes. Saliva samples are collected again, before and after the interaction task.

Before the experiments at the beginning (week 0) and end (week 8) of the study, a researcher interviews patients about their current OCD symptoms using the symptom checklist from the CY-BOCS and the distress level associated with each endorsed symptom on a scale from 0 to 10. The timeline for the experiments is depicted in Figure 2.

The experiment starts with a 5-minute resting period, in which the youth and parent are left in a room and are asked to relax and refrain from speaking and touching each other for 5 minutes. Patients complete an exposure task for up to 15 minutes led by a mental health professional in the research team in the presence of the parent unless the patient prefers to complete the exposure without the parent. The preparation and delivery of the exposure exercise were inspired by a treatment manual for youth with OCD [45] (page 73). The clinical researcher delivering the exposure provides psychoeducation about OCD and explains the procedure and rationale behind the exposure. During this ERP preparation phase, patients are asked to report their expectation for what will happen to their distress level during ERP with a multiple-choice question: (1) distress will increase until it is unbearable, (2) distress will remain the same, (3) distress will decrease over time. Throughout the exposure, patients are asked to report their level of distress.

Control dyads complete a physical activity task, in which they are shown an exercise dance video lasting approximately 10 minutes. The participants are not required to follow the video, but are asked to move their bodies in a way that increases their heart rate for the duration of the task. The physical activity task allows us to examine the performance of the biosensor under intense movement and measure salivary oxytocin reactivity following physical stress. In the final controlled condition, patient and control dyads complete a parent-child interaction task, in which they are asked to plan their best warm-weather (baseline) and cold-weather (follow-up) vacation together, inspired by previous work [46,47]. Saliva samples are collected from the youth and the parent after the resting period, exposure task, physical activity, and the parent-child interaction task.

For behavioral coding, the exposure and parent-child interaction tasks are video- and audio-recorded. Human coders will code the videos for interlocutor positive and negative affect, anxiety, arousal, affective touch, and synchrony, as well as parent sensitivity and youth engagement using the Coding Interactive Behavior manual [48].

Exposure sessions will also be coded for youth distress, avoidant/approach behavior, reassurance and proximity seeking, as well as parent accommodating behavior and discouraging avoidance, using items from the Exposure Rating Scale for Exposure and Response Prevention [49] and the Exposure Process Coding System [12,50]. Parent acceptance statements and confidence statements will be coded based on definitions in a treatment manual for OCD and anxiety disorders in youth targeting parents [51].

#### Observation Period (Weeks 1-8)

We ask participating youths and parents to wear the biosensor during their daily routines for 8 weeks from the time they wake up until the time they go to sleep.

Patients are asked to push the event tag button every time they feel stressed by their OCD symptoms. Their parents are asked to tag every time they notice their child is distressed by their OCD symptoms. Control youths are asked to push the button when they feel very scared. Control parents are asked to push the button when they notice that their child feels very scared. Researchers meet with participants up to twice a week during the 8 weeks of continuous data collection to exchange biosensors full of data with empty biosensors. A byproduct of having up to two weekly in-person meetings with participants to download biosensor data securely throughout the observation period is an implicit reminder to wear the biosensor.

#### Ecological Momentary Assessment

We are developing mobile apps for EMAs of child and parent emotional states to produce more data labels during the observation period. The Copenhagen Center for Health Technology Research Platform, on which the apps are built, is a research tool for data collection [52]. The child and parent apps contain information about the study and versions of the Positive and Negative Affect Questionnaire [53,54] that are triggered at random times during the day to offset the potential negative focus on OCD and anxiety symptoms from tagging these emotional events on the biosensor. In addition to reporting their emotional state, children and parents report whether they are in the company of the other, other family members, or friends to establish the context of EMA data, similar to previous studies [55]. The patient app also includes daily guided exposure tasks to help patients practice ERP outside of the clinic and a weekly form for reporting OCD symptoms and their severity.

### Study End Points

#### Defining Feasibility

The following study-specific feasibility criteria were developed: recruitment; retention rate; biosensor functionality; acceptability of the biosensor; compliance with wearing the biosensor; safety related to the biosensor; and physiological, audio, and visual markers of OCD. These are defined in Table 1.

**Table 1:** Operationalized feasibility success criteria.

Feasibility indicator			Criteria for success
Recruitment: consenting invited families (%)			*>*30% of invited families consent
Retention rate (%)			*>*80% retention
Biosensor functionality: unfixable malfunctions (N)			*<*10% irreparable malfunctions
Acceptability of biosensor: user experience questionnaire			*>*85% positive attitudes
**Compliance**			
	Days participants wear biosensor (N)	Biosensor worn *>*30% of days in study	
	Tagged events, falsely tagged events, missed events (N)	False events identified*; >*70% accurately tagged events	
Safety: adverse reactions to biosensor^a^			No major injuries or worsening of symptoms reported
Comparison of physiological signals as measured by the wearable biosensor; audio or visual signals; and reported, observed, or biochemical endpoints as markers of clinically relevant obsessive compulsive disorder parameters			>70% accuracy or high correlation with relevant endpoint

^a^Adverse reactions are defined as any negative effects of the biosensor. Investigators monitor adverse reactions at in-person visits, and they can be reported by parents, youths, or case workers.

#### Biosensor Experience

Children’s and parents’ level of physical (dis)comfort of wearing the biosensor, degree of satisfaction with the design, concerns of stigmatization, and data security as well as usability are assessed with a questionnaire in week 7. There are 13 multiple-choice questions answered on a 4-point scale (0=disagree a lot to 3=agree a lot) and one open-ended question. The English versions of the patient questionnaire and experience of biosensor questionnaire are provided in Multimedia Appendix 4.

Participants and parents are interviewed together after the experiments in week 8 about their experiences with the biosensor. They are also asked about their thoughts on the convenience and data security of using a mobile app for data collection (see Multimedia Appendix 4). At the end of the interview, participants are also asked whether they have any feedback about any aspect of their participation in the study.

#### Biosensor Compliance

Compliance to wearing the biosensor will be measured separately for youths and parents. Signals from the biosensor indicate whether the biosensor was worn while turned on. For the experiments, compliance is a binary outcome: either the participant wore the biosensor for the experiments or not. We will provide reasons for noncompliance (eg, discomfort or technical problems with the biosensor). For the observational period, we will count the number of days the participants wore the biosensor and define adherence as more than 30% of days (1 day=at least 3 hours) in 1 month of participation. The biosensor provides time data per recording session in hours, minutes, and seconds, and provides the dates on which the sessions were recorded. We will also report total time in hours that each participant wore the biosensor.

#### OCD Markers

##### Distress

During the lab visits, OCD distress is rated by youths in two ways. First, OCD distress related to each current OCD symptom is rated before ERP sessions on a symptom hierarchy on a scale from 0 (no distress) to 10 (maximum distress). During ERP sessions, the clinical researcher asked youths to rate their current distress levels after 1, 3, 5, 7, 10, and 15 minutes on the same scale as the symptom hierarchy. When OCD distress is rated during an ERP session, it is called subjective units of distress (SUDs) [56].

During the in-the-wild observational period, OCD distress is a binary measure, with patients and their parents marking events of OCD distress. Control dyads mark occurrences of youth fear to compare clinical to subclinical distress.

##### ERP Quality

Two major theories for mechanisms of change in ERP include emotional processing theory and inhibitory learning theory. According to emotional processing theory, ERP works by replacing an association between a stimulus and a feared outcome with a new harmless association [57,58]. Here, quality ERP sessions require habituation, defined as sessions in which distress levels decrease from the first measurement until the last measurement, and the decrease in distress occurs in the absence of avoidance, reduced task difficulty, and accommodation as defined previously [12]. This theory also assumes that there is an optimal level of distress that is neither too low nor too high for conducting effective exposures [59]. As subjective distress may not correspond to physiological levels of arousal, we will use both measures. Using SUDs and physiological measures of arousal, we will categorize low, medium, and high distress.

Alternatively, the inhibitory learning theory states that successful exposures are characterized by reduction in expectancy of an aversive outcome by creating alternative harmless associations that compete with the feared expectations [60]. A therapeutic expectancy violation occurs when an anticipated negative outcome fails to actualize and a benign or positive outcome occurs instead [60,61]. We will calculate distress-related expectancy violations in the baseline and follow-up exposure sessions in three ways. First, an expectancy violation occurs when the patient’s expected distress trajectory as reported in the multiple-choice question before an ERP does not match the actual distress trajectory during ERP. Another way that we will define expectancy violation is by subtracting the actual maximum distress level during ERP by the distress level reported prior to an ERP session on the symptom hierarchy [62]. The final way that we will define an expectancy violation is by subtracting the distress level reported prior to ERP on the symptom hierarchy by the actual distress level at the end of an ERP session [62].

##### OCD Symptom Severity

The CY-BOCS, the gold-standard semistructured clinical interview for assessing OCD symptom severity in youths on a scale from 0-40 [63], is completed at baseline and at week 8 of follow-up. Items 1-10 are summed to obtain a total OCD severity score, with higher scores indicating higher severity. Items 1-5 can be summed to obtain an obsession severity score and items 6-10 can be summed to obtain a compulsion severity score. Change in OCD severity is calculated by subtracting the week 8 CY-BOCS score from the baseline CY-BOCS scores. Remission is defined as a 30% or greater reduction in the CY-BOCS score from baseline to follow-up. The CY-BOCS also contains information about types of OCD symptoms, such as those marked by anxiety or disgust. We expect these two mental states to have different effects on physiological signals as previously found [64].

##### Quality of Life

KIDSCREEN-52 is a parent- and child-rated questionnaire of child (8-18 years) health-related quality of life [39]. Higher scores indicate a better quality of life. In this study, we are particularly interested in the following subscales: psychological well-being, mood and emotions, autonomy, and parent relation and home life. These subscales provide information about the internal states of patients and controls as well as family dynamics.

##### Family Interactive Processes

The FAS-PR is completed by parents of patients and asks parents to rate how often they modify their routines due to their children’s OCD and how frequently they help their children complete OCD rituals [43]. One item assesses how much distress the parents experience because of their accommodating behavior. Three items ask parents to rate how unhappy, anxious, angry, or violent their children become if not accommodated [44].

To obtain insight into the family interactive characteristics of patients and controls, we ask all parents to complete the following subscales on the FES-PR: cohesion, expressiveness, conflict, independence, organization, and control. The FES-PR assesses the rater’s current perception of their family environment [65].

The PSS contains 18 items that ask parents to rate how stressful they experience parenting their participating child [40]. Parents rate the extent to which they disagree or agree on a 5-point scale (1=highly disagree to 5=highly agree) to statements about their role as parents.

##### Parent Affective Symptoms

Parents report their symptoms of depression, anxiety, and stress on the 42-item DASS [41,66,67]. These internal states of parents will likely affect their physiological signals.

##### Oxytocin

Oxytocin levels are measured in youths and their parents before and after the controlled conditions during lab visits, as described in the Experiments section above. Salivary oxytocin response is defined as a change in oxytocin levels measured before and after a condition (physical activity, exposure, parent-child interaction). We ask participants to refrain from eating 2 hours and drinking 30 minutes before collecting the first saliva sample. To collect one saliva sample, participants are asked to chew on a Salivette (Sarsted, Rommelsdorf, Germany) for 60 seconds. The saliva samples are frozen at –20°C. At the biochemistry laboratory, the samples are centrifuged twice with a 2-day interval at 4°C at 1500 ×*g* for 20 minutes. The obtained liquid samples are lyophilized at –80°C/0.02 mbar overnight and stored at –20°C. On the day of the assay, the freeze-dried samples are reconstituted with assay buffer. The factor depends on the amount of saliva before freeze-drying. Samples are concentrated three times. The immunoassay is performed with the Enzo (NY, USA) oxytocin kit or a kit of comparable detection sensitivity. Measurements are performed twice, and the sample concentrations are calculated using WorkOut version 2.5 software that is provided with the enzyme-linked immunoassay reader according to relevant standard curves.

Measuring oxytocin in saliva is a noninvasive and easy way of estimating peripheral oxytocin levels [68]. Salivary oxytocin is sensitive to manipulations of stress and social interaction, as demonstrated by increases in oxytocin levels after 10 minutes of physical exercise [69], anticipation of and engagement in a socially stressful condition [70], and a 7-minute parent-child interaction task [71]. Higher oxytocin levels have been associated with parental synchrony and affect synchrony between parents and infants [72]. Higher oxytocin levels have also been linked to lower heart rate [69,73].

##### Synchrony

Synchrony is operationalized as two processes that are or become correlated above chance levels [74]. We will calculate synchrony between youth and parent affect, behavior, oxytocin, and physiological signals.

### Data Analysis

#### Analysis Plan

We will summarize the demographic and clinical characteristics of the patients, control youths, parents of patients, and parents of control youths at baseline. At baseline, week 4, and week 8, we will summarize parental stress for patients and controls and family accommodation for patients in plots. At baseline and week 8, we will summarize child quality of life for patients and controls and OCD severity for patients in plots. We will also summarize physiological signals, accelerometer data, facial emotion recognition, movement, speech, and observational codes under the controlled conditions (rest, exercise, exposure, parent-child interaction) separately. Physiological and accelerometer data collected in the wild will be summarized in a plot with time on the x-axis, separately for patients and controls. Means with SDs will be reported for continuous variables and frequencies and proportions will be reported for categorical variables. We will not perform imputations of missing data. Data will be analyzed on an intention-to-treat basis regardless of protocol violations.

#### Model Development and Testing

Detailed statistical/ML analysis plans will be published separately. Table 2 provides an overview of the primary prediction targets and the hypothesized associations among signals and clinical endpoints. As a first step in learning the associations between signals and OCD-related parameters, we will plot the progression of endpoints (responses for future paired *t* tests) and use mixed effects models with repeated measures to assess associations between oxytocin, individual biosignal features, and outcomes such as stress-rest and case-control comparisons. Confounding factors and important interactions will be included to the extent possible. These initial statistical results will mainly serve to estimate sample sizes for future studies. Substudies will focus on assessing automating behavioral observations using audio and video data from parent-child interactions and exposure sessions.

We will test the feasibility of developing AI tools that use signals from a wearable biosensor and audio and video recordings of human interactions. Audio and video recordings are collected at two time points for each participant dyad, which will increase power of these analyses. Furthermore, the exposure tasks last for over 20 minutes and will be sliced into different events: ERP preparation, ERP, ERP debrief. These events will be segmented in 5-minute slices for analyses, which will increase the number of observations.

We will use ML models for classification and prediction of OCD-related and physical stress, as well as prediction of in-the-wild distress episodes. We will use a double-loop cross-validation for model selection and to assess model accuracy within and across subjects. Differences in accuracy within and across subjects can provide information on the needed number of observations for developing robust models in the future. We will use pretrained speech emotion recognition models and computer vision models to estimate behavioral labels from audio and video, respectively.

**Table 2:** Overview of clinically relevant parameters and hypothesized associations among signals and clinical endpoints.

Prediction target	Definition	Physiological signals	Paralinguistic signals	Linguistic signals	Visual/behavioral signals	Biochemical signals	Ground truth/validation
OCD^a^ distress (youth)	Stress, fear, worry, disgust, embarrassment, shame, anger, sadness caused by obsessions/compulsions	Change in HR^b^ depending on emotion; increased EDA^c^ and temperature	High activation; negative valence	Negative emotion statements	Negative emotion facial expression; increased movement	Low oxytocin	Biosensor tags; (SUDs^d^); child negative emotions (CIB^e^); avoidance (ERP^f^); child stress (FAS^g^); consequences (CY-BOCS^h^)
OCD severity	Time spent on, interference, and distress caused by obsessions/compulsions; resistance of and control over obsessions/compulsions	Change in HR depending on emotion; increased EDA and temperature	High activation; negative valence	Negative emotion statements	Negative emotion facial expressions	Low oxytocin	Symptom hierarchy; child negative emotions (CIB); avoidance (ERP); mood and emotion (KIDSCREEN-52)
OCD distress (parent)	Stress, embarrassment, worry, shame, anger, sadness caused by youth’s OCD	Change in HR depending on emotion; increased EDA and temperature	High activation; negative valence	Distress statements	Negative emotion facial expressions	Low oxytocin	Biosensor tags; parent negative emotions (CIB); parent stress (FAS); parental stress (DASS^i^)
Overdemanding parenting [51]	Expecting youth not to feel distressed despite lived experience [51]	HR, HRV^j^, EDA; lower resting HRV; asynchrony	High activation; negative valence; asynchrony; interruptions	Example: “You must…”	Negative emotion facial expressions	Low oxytocin; low concordance between parent and child	Intrusiveness, criticism, low validation (CIB); conflict (FES^k^); low demand (FAS, item 5)
Overprotective parenting [51]	Cognitions/behaviors aimed at protecting child from distress or harm [51]	HR, HRV, EDA; lower resting HRV; asynchrony	Positive valence; asynchrony	Protective statements	Proximity; affective touch	Low oxytocin; low concordance between parent and child	Accommodation (ERP); participation and modification (FAS); autonomy (KIDSCREEN-52)
Supportive parenting [51]	Nonjudgmental, emotional and cognitive, empathy and conviction that child can cope [51]	Higher resting HRV; HR, HRV, EDA; synchrony	Positive valence; entrainment [18]	Accepting and confidence statements [51]	Movement synchrony	High oxytocin; high concordance between parent and child	Youth engagement, parent sensitivity (CIB), synchrony (ERP); discourage avoidance (FAS low score); cohesion (FES); parent relation (KIDSCREEN-52)

^a^OCD: obsessive compulsive disorder.

^b^HR: heart rate.

^c^EDA: electrodermal activity.

^d^SUD: subjective unit of distress.

^e^CIB: coding interactive behavior [48].

^f^ERP: exposure and response prevention.

^g^FAS: family accommodation scale [43,44].

^h^CY-BOCS: Children’s Yale-Brown Obsessive Compulsive Scale.

^i^DASS: depression anxiety and stress scale [41].

^j^HRV: heart rate variability.

^k^FES: family environment scale [42].

#### Feasibility Outcomes

Study-specific feasibility criteria are defined in Table 1. Where appropriate, we will provide 95% CIs. Feasibility outcomes are binary: “success” indicates that the a priori feasibility criteria have been met, whereas “revise” indicates that the criteria have not been met. “Success” will indicate that no or small changes are needed before proceeding with a larger study and “revise” will indicate the need for major changes before proceeding.

We will also identify the clinical measures needed to validate the AI tools. Measures will be retained for further studies if they are comprehensible to the participants, do not overburden participants, and can be practically delivered to participants without overburdening research staff. EMAs about emotions should not exceed 60 seconds to complete. Questionnaires and semistructured interviews about participant experiences with the biosensor will be used to generate hypotheses that can be tested in a larger study and, if relevant, to improve researcher scripts for introducing the biosensor to participants. We will report frequencies of responses to the user experience questionnaires.

### Dissemination

Study results will be released to participants and referring clinicians upon request. Results will be published in scientific journals and conferences targeting mental health professionals and researchers within computer science. We follow authorship guides set by the National Academies of Sciences, Engineering, and Medicine [75] and the International Committee of Medical Journal Editors [76].

## Results

The first participant was enrolled on December 3, 2021, and recruitment closed on December 31, 2022. Nine patient dyads and nine control dyads were recruited. Sixteen participating dyads completed follow-up assessments. We expect to submit results of our study by the summer of 2023. 

## Discussion

### Study Overview and Significance

One percent of children and adolescents will be diagnosed with OCD before their 18th birthday [77]. Today, diagnosis and severity ratings depend on self-report and clinical observation, and no objective measures exist for OCD symptoms. ML models that detect distress levels would automate clinical severity monitoring and passively collect objective measures allowing therapists to adjust exposure treatment accordingly. Automatic, continuous distress detection may also facilitate the monitoring of treatment response across therapy sessions as well as monitoring disorder progression in individuals with subclinical symptoms. Finally, ML models that detect changes in mental states would facilitate increasing awareness of connections between thoughts, emotions, behaviors, and bodily sensations, forming an essential part of effective therapy [13].

In this protocol, we present the study design and methods of a pilot study that will evaluate the feasibility of using a commercially available wearable biosensor to monitor symptoms of OCD in youth from physiological signals. Embedded in our pilot longitudinal, case-control study are substudies of audio/visual signals and actively collected biochemical parameters from youths and parents with the goal of detecting and predicting episodes of OCD-related distress.

Potential feasibility issues involve recruiting and retaining participants, biosensor safety and functionality, participant adherence to wearing the biosensor, and logistics of collecting and processing data. The size and design of the biosensor and the lack of a substantial clinical intervention may deter participation. User experience questionnaires and interviews explore themes of stigma attached to wearing a biosensor. A previous study demonstrated that a group of 16 youth, aged 10 to 17 years, participating in a study of similar length wore a similar biosensor for an average of 11 out of 30 days and one child managed to wear the wristband 5 days per week [78]. The study aimed to develop a personalized weight management program with the E3 wristband and asked youths to wear the biosensor for at least 5 days per week for 3 months [78]. No technical difficulties were reported for the wristband [78]. Youth in the wristband condition reported a similar level of (dis)comfort as youth in the control condition [78]. Nonetheless, the researchers speculated as to whether the youths did not wear the wristband due to concerns that it would attract unwanted attention from peers [78]. Some participants expressed concern of being surveilled by the biosensor [78]. To address potential concerns in our participants, we will thoroughly explain what the wristband is and is not capable of and coach the participants in informing others about their new accessory. The E4 wristband has been used to monitor arousal in 15 children as young as 2 years with Down syndrome during 7-minute parent-child interaction sessions [15]. EDA data from children were deemed useable (90% of interactions tracked) for 27 out of 30 interactions; from parents, EDA data were useable for 23 out of 30 interactions [15]. Missing data were due to 7 instances of software malfunctions [15].

A byproduct of having up to two in-person meetings with participants to download biosensor data securely throughout the observation period may be an implicit reminder to wear the biosensor. We will examine whether participants were more likely to wear the biosensor on data collection days. Asking youths with obsessions, compulsions, and safety behaviors to tag OCD-related events may interact with or become a symptom. This theme is also explored in the user experience interview.

### Strengths and Limitations

The strengths of the protocol include the ecological validity of in-the-wild passively collected physiological signals; collecting physiological signals under repeated controlled conditions; the case-control design; and supplementing with multimodal signals from audio, video, and biochemistry. One limitation of the protocol is the small sample size. The sample size limits generalizability of the statistical and ML results. The ML models risk producing false-positive predictions. In the case of high individual variation, false negatives will also be a risk. Nonetheless, we have enough observations to evaluate the feasibility of making the proposed predictions. We have up to 8 weeks of physiological signal data per participant. Moreover, previous studies indicate that these physiological signals are sensitive to strong sustained stressors [75] such as those experienced in OCD.

### Conclusion

If the findings of this pilot study support our hypotheses of sufficient feasibility, subsequent larger studies will further develop and test ML models that can detect and predict OCD severity, distress, and related family processes that maintain OCD. Ultimately, these innovations will contribute to increasing knowledge about objective markers of OCD severity and OCD-related, clinically relevant family processes. Furthermore, the AI tools we aim to develop have the potential to assist in validation of assessments of the severity of psychiatric disorders and inform treatment decisions. Such AI tools would enable monitoring disease and treatment planning. For example, automatic clinical severity classification models could be applied in the therapy room or in the patients’ everyday lives while youths are on treatment wait lists to monitor symptoms or between therapy sessions to monitor and support treatment progress.

## Appendix

Multimedia Appendix 1 SPIRIT Checklist.

Multimedia Appendix 2 Model consent form.

Multimedia Appendix 3 Assessment timeline.

Multimedia Appendix 4 Sample questionnaires and interviews.

## Abbreviations

AI: artificial intelligence
CONSORT: Consolidated Standards of Reporting Trials
CY-BOCS: Children’s Yale-Brown Obsessive-Compulsive Scale
DASS: Depression Anxiety Stress Scales
EDA: electrodermal activity
EMA: ecological momentary assessment
ERP: exposure and response prevention
FAS-PR: Family Accommodation Scale- Parent Rated
FDA: Food and Drug Administration
K-SADS-PL: Schedule for Affective Disorders and Schizophrenia for School-Age Children-Present and Lifetime Version
ML: machine learning
OCD: obsessive compulsive disorder
PPG: photoplethysmography
PSS: Parental Stress Scale
REDCap: Research Electronic Data Capture
SPIRIT: Standard Protocol Items: Recommendations for Interventional Trials
SUD: subjective unit of distress
TECTO: Treatment Effects of family based Cognitive Therapy in children and adolescents with Obsessive compulsive disorder

## Data Availability

After results are published, we will strive to make a depersonalized data set publicly available in a legally compliant way. We will open-source the machine learning code where relevant in conjunction with publications.
