# Supplementary material for: A Wearable Artificial Intelligence Feedback Tool (Wrist Angel) for Treatment and Research of Obsessive Compulsive Disorder: Protocol for a Nonrandomized Pilot Study
Source: JMIR Res Protoc. 2023 Jul 24;12:e45123. doi: 10.2196/45123 (PMC10407771; doi:10.2196/45123)
Supplement: Multimedia Appendix 2 [file resprot_v12i1e45123_app2.pdf]

[The following text is from our RedCap electronic consent form]

Consent to Parental Participation – Patient

Below, you can give consent for participation as a parent in a health science research project, Wrist Angel.

FOR PARENTS OF PARTICIPANTS - PARENT PARTICIPATION
INFORMED CONSENT FOR PARTICIPATION IN A HEALTH SCIENCE RESEARCH PROJECT

Research project title: Wrist Angel: A Wearable Artificial Intelligence Feedback Tool for OCD Treatment and research

Statement from the participant:
I have received written and oral information, and I know enough about the purpose, method, advantages, and disadvantages of the project to give my consent.
I know that participation is voluntary and that I can always withdraw my consent without losing my current or future rights to treatment.
I give consent to participate in the research project and have received a copy of the written information about the project.

It is possible to obtain/download a copy of this consent form once the consent form has been signed.
I also consent to the following, in connection with the project:

	Yes	No
1) recording video of me		
2) recording audio of me		
3) providing saliva samples, which will be stored in a research biobank		

Name: ____________________
Date: ___________

[Participating parent] signature ___________________________

Do you wish to receive information about the results of the project?

- ○ yes
- ○ no

Declaration by the researcher who provided information about the project:

I declare that the participant has received oral and written information about the study.
In my opinion, sufficient information has been given the participant to make a decision about participating in the research project.

The name of the researcher who submitted the information: _____________________
Date: ____________
[Researcher] signature: ______________________________
