# Supplementary material for: A Wearable Artificial Intelligence Feedback Tool (Wrist Angel) for Treatment and Research of Obsessive Compulsive Disorder: Protocol for a Nonrandomized Pilot Study
Source: JMIR Res Protoc. 2023 Jul 24;12:e45123. doi: 10.2196/45123 (PMC10407771; doi:10.2196/45123)
Supplement: Multimedia Appendix 3 [file resprot_v12i1e45123_app3.pdf]

Assessments and participant timeline.

| | **Baseline**<br>**Lab visit** | **Week 1** | **Week 2** | **Week 3** | **Week 4** | **Week 5** | **Week 6** | **Week 7** | **Week 8**<br>**Lab visit** |
|---|---|---|---|---|---|---|---|---|---|
| Demographic information | x | | | | | | | | |
| OCD symptom severity | x | | | | | | | | x |
| OCD symptom hierarchy | x | x | x | x | x | x | x | x | x |
| OCD SUDS | x | | | | | | | | x |
| Quality of life - youth | | x | | | | | | | x |
| FAS | | x | | | x | | | | x |
| FES | | x | | | | | | | |
| PSS | | x | | | x | | | | x |
| Parent affective symptoms | | x | | | | | | | |

| | | | | | | | | | |
|---|---|---|---|---|---|---|---|---|---|
| Exposure form | x | | | | | | | | x |
| Experiments with oxytocin measurements | x | | | | | | | | x |
| Biosensor user experience questionnaire | | | | | | | | x | |
| Biosensor user experience interview | | | | | | | | | x |
| Record adverse events | x | x | x | x | x | x | x | x | x |

Note. SUDS and exposure forms should be available daily. OCD: obsessive compulsive disorder; SUDS: subjective units distress.
