# Supplementary material for: A Wearable Artificial Intelligence Feedback Tool (Wrist Angel) for Treatment and Research of Obsessive Compulsive Disorder: Protocol for a Nonrandomized Pilot Study
Source: JMIR Res Protoc. 2023 Jul 24;12:e45123. doi: 10.2196/45123 (PMC10407771; doi:10.2196/45123)
Supplement: Multimedia Appendix 4 [file resprot_v12i1e45123_app4.pdf]

Supplementary material

Sample questionnaires and interviews

1. Demographic questionnaire pages 1-3
2. Hormone questionnaire for parents pages 4-5
3. Biosensor experience questionnaire page 6
4. Biosensor experience interview pages 7-8

1. Demographic questionnaire

Background information (Parent)

Please complete the questionnaire below.

Thank you in advance!

Date of completion: ______________

1. I am the child's:
   - o Biological parent
   - o Other

1a. If "other", specify: ____________________

2. Gender: I am
   - o A woman
   - o A man
   - o Transgender
   - o Other
3. Where was your child born?
   - o Denmark
   - o Other

3a. If "other", please specify where your child was born: _____________

4. Is your child a Danish citizen?
   - o Yes
   - o No

4a. If "no", specify your child's nationality: ____________________

5. Where was your child's mother born?
    - o Denmark
    - o Other

5a. If "other", please specify where your child's mother was born: _____________

6. Is your child's mother a Danish citizen?
    - o Yes
    - o No

6a. If "no", specify your child's mother's nationality: ____________________

7. Where was your child's the father born?
    - o Denmark
    - o Other

7a. If "other", please specify where your child's mother was born: _____________

8. Is your child's father a Danish citizen?
    - o Yes
    - o No

8a. If "no", specify your child's mother's nationality: ____________________

9. What is your child's mother's highest completed level of education?
    - o Pre-education (kindergarden)
    - o Primary education (1st – 6th years)
    - o Primary education (7th-10th year)
    - o Upper secondary education (10th year)
    - o Upper secondary education (11th – 12th years)
    - o Short cycle higher education (13th – 14th years)
    - o Vocational bachelor education (15th – 16th years)
    - o Master's programs (17th – 18th years)
    - o PhD programs (19th year)
    - o None of the above

9a. If the answer above is "none of the above", please specify. _____________

10. What is your child's father's highest completed level of education?

10a. If the answer above is "none of the above", please specify. _____________
    - o Pre-education (kindergarden)
    - o Primary education (1st – 6th years)
    - o Primary education (7th-10th year)
    - o Upper secondary education (10th year)
    - o Upper secondary education (11th – 12th years)

- Short cycle higher education (13th – 14th years)
- Vocational bachelor education (15th – 16th years)
- Master's programs (17th – 18th years)
- PhD programs (19th year)
- None of the above

1. Hormone questionnaire for parents

Hormones (Parents)[1]

Please complete the questionnaire below.

Thank you in advance!

Date of completion: ______________

1. Do you take medicine with hormones? (for example melatonin, estrogen, testosterone, growth hormone, thyroid hormone)
   a. Yes
   b. No
2. What is/ are the name(s) of the medication(s)? ________________________
3. How do you take the medicine?
   a. Pill
   b. Injection
   c. Other
4. How often do you take the medication? (for example every day, once per day, once a month) ________________
5. Dosis (enter in mg, µg, for example) ____________________
6. Have you ever menstruated?
   a. Yes
   b. No
7. Have you started menopause?
   a. Yes
   b. No
8. When did you approximately have your last regular menstruation?
   a. Yes
   b. No
9. When was the first day of your latest menstruation? _______________________________
10. Do you use contraception with hormones? (for example birth control pills, hormonal IUD)
    a. Yes
    b. No
11. What is the name of the contraceptive? __________________________
12. Are you pregnant?
    a. Yes
    b. No
    c. Do not know
13. Which pregnancy week are you currently in?
    a. Week 1-12
    b. Week 13-26

   c. Week 27-40
   d. Week 41+
14. Are you nursing?
   a. Yes
   b. No

[1] Please note that the child questionnaire is similar but excludes questions about menopause. Also, in the electronic system some questions only appear if certain conditions are met, based on answers to previous questions, so that the participant only sees questions that are relevant.

3. Biosensor experience questionnaire

User experience: wristband with biosensor - Patient

Participant ID ________ Date: __________

We would like to know what it was like for you to wear the wristband.

Please read each statement and chose the number (0, 1, 2, or 3) that best describes how you feel.

	0<br>Strongly disagree	1<br>Slightly disagree	2<br>Agree	3<br>Strongly agree
I like how the wristband looks.				
The wristband looks too big.				
I was embarrassed to wear the wristband.				
The wristband looks cool.				
The wristband attracted too much attention.				
The wristband was comfortable.				
The wristband fit well around my wrist.				
The wristband was easy to use.				
The wristband was easy to charge.				
I often forgot to wear the wristband.				
I wanted to wear the wristband.				
It was irritating to push the button on the wristband.				
I remembered to push the button on the wristband every time OCD was bothering me.				

Is there anything else you would like to tell us about your experience wearing the wristband?

____

____

4. Biosensor experience interview

Biosensor & App user experience interview – Patient (Researcher form)

Participant ID: _____________

Wrist Angel end of study Semi-structured Interview

The child and parent can be interviewed together but be aware of things that the child or parent may not want to say in front of the other.

1. What would you think about using an app to collect data in this study? (child) ________________________________
2. What would you think about using an app to collect data in this study? (parent) _______________________________
3. How would you feel about sharing personal information with a research app? (child) ________________
4. How would you feel about sharing personal information with a research app? (parent) ________________
5. Is there anything that you would like to tell us about your experiences with the biosensor? (child) ________________
6. Is there anything that you would like to tell us about your experiences with the biosensor? (parent) ________________
7. How easy was it for you to use the symptom hierarchy? (patient) (Note to interviewer: you may need to remind the patient what the symptom hierarchy is. You can show them an example.)
   - Very difficult
   - Difficult
   - easy
   - very easy
8. Can you say more about your experiences with using the symptom hierarchy? (patient and parent) _______________________
9. How easy was it for you to use the exposure practice form? (patient) (Note to interviewer: you may need to remind the patient what the symptom hierarchy is. You can show them an example.)
   - Very difficult
   - Difficult
   - easy
   - very easy
10. Can you say more about your experiences with using the exposure practice form? (patient and parent)

____

11. Would you prefer to write your symptoms on paper or use an app? (patient)
12. Was there any information from the study that you wanted to share with your clinician? (patient)
    a. Not at all
    b. No
    c. Yes
    d. Very much
13. What information that we collected in the study would you have liked to share with your clinician?
    a. Nothing
    b. Symptom hierarchy
    c. Exposure practice form
    d. Feelings
    e. Everything
14. To what end would you want to share this information with your clinician (patient) ____________
15. If you had problems with the biosensor, could you get the help you needed? (elaborate prompt)
16. Is there anything else that you think we should know about the biosensor or the study in general?
